# Factors Associated with Refusal or Discontinuation of Treatment in Patients with Bladder Cancer: A Cohort Population-Based Study in Taiwan

**DOI:** 10.3390/ijerph18020618

**Published:** 2021-01-13

**Authors:** Nai-Tan Chang, Ying-Hsu Chang, Yu-Tung Huang, Shu-Ching Chen

**Affiliations:** 1 Department of Nursing, Chang Gung Memorial Hospital at Linkou, Taoyuan 333, Taiwan; nandan@cgmh.org.tw; 2 School of Nursing, College of Medicine, Chang Gung University, Taoyuan 333, Taiwan; 3 Department of Urology, New Taipei Municipal Tucheng Hospital Chang Gung Memorial Hospital, New Taipei 236, Taiwan; anatomy@cgmh.org.tw; 4 Division of Urology, Department of Surgery, Chang Gung Memorial Hospital at Linkou, Taoyuan 333, Taiwan; 5 Department of Medicine, College of Medicine, Chang Gung University, Taoyuan 333, Taiwan; 6 Center for Big Data Analytics and Statistics, Chang Gung Memorial Hospital at Linkou, Taoyuan 333, Taiwan; anton.huang@gmail.com; 7 School of Nursing and Geriatric and Long-Term Care Research Center, College of Nursing, Chang Gung University of Science and Technology, Taoyuan 333, Taiwan; 8 Department of Radiation Oncology and Proton and Radiation Therapy Center, Chang Gung Memorial Hospital at Linkou, Taoyuan 333, Taiwan

**Keywords:** bladder cancer, refusing treatment, discontinuing treatment, patient non-compliance, case management

## Abstract

Cancer treatment causes adverse effects that lead to refusal or discontinuation of treatment. The purposes of this study were to identify 1) the factors associated with and 2) the reasons for refusing and discontinuing treatment in patients with bladder cancer (BC). We conducted a retrospective cohort study in patients diagnosed with BC in Taiwan from 1 January 2014 to 30 June 2019 using a linked cancer registry database. Of the 1247 BC patients in the study cohort, 2.1% reported refusing treatment. Patients with less education and those diagnosed at cancer stage II–IV were more likely to refuse treatment. The major reason for refusing treatment was “patient or the family considered patient’s poor physical condition (chronic disease or unstable systemic disease), difficulty in enduring any condition likely to cause physical discomfort from disease treatment”. A total of 4.3% of BC patients reported discontinuing treatment. Patients not living in the northern region of Taiwan and those diagnosed at cancer stage II–IV were more likely to terminate treatment before completion. The major reason given for discontinuing treatment was inconvenient transportation. Sufficient social resources and supportive care can help BC patients cope with the physical and psychological burden of treatment.

## 1. Introduction

Cancer is a leading cause of mortality worldwide, with bladder cancer (BC) the 10th most common cancer worldwide and the seventh most common in men, with an estimated 430,000 new cases diagnosed every year [1,2]. In Taiwan, BC represents the ninth most frequent cancer in males, with approximately 1700 incident cases per year [3]. Occupational exposure, tobacco smoking, and age all increase the risk of BC, a cancer with a high recurrence rate and progression that requires repeated therapies [4].

Surgery alone or combined with chemotherapy (CT) or concurrent chemoradiation therapy (CCRT) are the major treatments for BC [4]. Adverse effects associated with these treatments include temporal or permanent changes in bladder function (such as bladder irritation, burning during urination, urgency to urination, frequency urination, and cystitis), nausea, vomiting, poor appetite, fatigue, and psychological distress [5]. These conditions are associated with refusing and discontinuing treatment [6], and may result in rapid deterioration, distressing physical symptoms, metastasis, and even a lower likelihood of survival [7].

Refusing treatment refers to patients declining to receive standard therapy within four months of receiving treatment recommendations [8]. Approximately 3.54–24.2% of cancer patients reported refusing or avoiding medical treatment [7,8,9,10]. Refusing treatment is associated with older age [7,8,10], low educational status [7], being unmarried [10], advanced stage disease [8], low body weight [7], poor physical condition [9], lower performance status [7], hearing negative treatment experiences from family or friends [9], and poor family support [9].

Discontinuing treatment is defined as a patient’s unplanned discontinuation of scheduled treatment more than 90 days after prior treatment cycles [11]. Some 6–57.6% of cancer patients discontinue treatment [12,13,14,15]. The factors associated with treatment regimen termination include older age, comorbidity, treatment toxicity [12,16,17], lack of transportation [9,18], impairment in activities of daily living [15], poor physical performance [13,17], advanced stage disease [16,17], and long treatment courses [17].

Although previous studies have explored these issues, most research has focused on patients diagnosed with breast cancer [8,12,13,14], lung cancer [7,10], colorectal cancer [9], head and neck cancer [18], and prostate cancer [15]. Along with unhealthy lifestyle (e.g., smoking, hair dyeing, and other contacts with dyes) and contact with industrial pollutants (e.g., rubber, chemicals used in medicine, printing, and other industrial solvents), it is important to understand why patients refuse or discontinue treatment and how these gaps in medical treatment relate to each other. Based on the literature review, we assumed that BC patients may refuse or discontinue treatment based on their personal characteristics (older age [7,8,10], marital status [10], physical performance [7,13,15,17], and support system [9,18]) or clinical characteristics (cancer stage [8], previous experiences [9], comorbidity, the side effects of treatment, and treatment protocol [12,16,17]). Patients diagnosed with BC may also decline the physician recommended treatment plan and risk discontinuing treatment during active therapy, an important topic to which insufficient attention has been devoted. This study aimed to (1) identify the factors (demographic: age, marital status, education level, employment status, and living location; and clinical characteristics: cancer stage, recurrence or not, and types of treatment) associated with refusing or discontinuing treatment and (2) examine the reasons for refusing or discontinuing treatment in patients with BC.

## 2. Materials and Methods

### 2.1. Design and Population

#### 2.1.1. Design

This study was a retrospective cohort secondary analysis of population-based data to investigate the factors and reasons associated with refusal or discontinuation of treatment in BC patients.

#### 2.1.2. Study Subjects

The inclusion criteria were: (1) age of 20 years or more, (2) diagnosis of non-muscle invasive bladder cancer (NMIBC) or muscle invasive bladder cancer (MIBC) (the International Statistical Classification of Diseases and Related Health Problems, 10th revision—ICD-10. Codes C670-679) [19], (3) patient receipt of case management within a year after a cancer diagnosis, and (4) receipt of active anti-cancer treatment at the study institution. The final population sample for analysis was 1247 after excluding patients with missing data and those aged less than 20 years.

### 2.2. Data Resources

The data were taken from the Cancer Registry Database (CRD) of a medical center in Northern Taiwan, the main cancer institute in Taiwan, for the period 1 January 2014 to 30 June 2019. Diseases were defined based on ICD-10 codes [19]. Cancer diagnosis was based on ICD-10 codes as determined by clinical findings; therefore, the cancer diagnosis in this study should be accurate and reliable.

### 2.3. Ethical Considerations

The study was approved by the Institutional Review Board of Chang Gung Medical Foundation, and a permission certificate (Number: 201900232B0) was obtained. All identifying information in the population-based dataset was deleted before data analysis, and the review board waived the requirement for signed informed consent.

### 2.4. Measurements

#### 2.4.1. Checklist of Reasons for Refusing Treatment and Discontinuing Treatment

A checklist related to reasons for refusing or discontinuing treatment was derived from interviewing patients who had refused treatment or discontinued treatment, chart records, previous studies [9,20], and clinical observation. The checklist contained 13 items: (1) patient or the family considered the patient’s poor physical condition (chronic disease or unstable systemic disease), or difficulty in enduring any condition likely to cause physical discomfort from disease treatment (patient’s poor physical condition); (2) inconvenient transportation; (3) disease progression; (4) distrust of physician ability and skills; (5) patient or their family or friends experienced negative treatment effects and worried about the side effects of treatment; (6) poor family support; (7) the patient’s old age; (8) economic difficulties; (9) selected complementary and/or alternative medicine; (10) changed treatment plan by physician; (11) longer awaiting time for treatment; (12) dissatisfaction with medical care; and (13) death. The content validity of the checklist was evaluated by experts who determined that the content was satisfactory. The results of responses to the checklist of reasons for refusing or discontinuing treatment were recorded and assessed by a primary case manager at diagnosis, at follow up during treatment, and at one year after diagnosis.

#### 2.4.2. Demographic and Clinical Characteristics Form

Demographic characteristics included age, gender, marital status, educational level, occupational status, and region of residence. Clinical characteristics included diagnosis, cancer stage, recurrence, medical treatment, treatment refused, and treatment discontinued.

### 2.5. Statistical Analysis

Data were analyzed using SPSS 26.0 (IBM Corp., Inc., Armonk, NY, USA). Demographic characteristics, clinical characteristics, and reasons for refusing or discontinuing treatment were described using frequency distributions, percentages, means, and standard deviations (SD), as appropriate. Logistic regression analysis was used to identify the factors associated with refusing or discontinuing treatment (dependent variable). Independent variables included age (≤64 vs. ≥65 years), education level, marital status (unmarried vs. married), employment status (no vs. yes), region of residence in Taiwan (Northern Taiwan vs. other), cancer stage (0–I vs. II–IV), recurrence (no vs. yes), surgery (no vs. yes), and CCRT (no vs. yes).

Odds ratio refers to the ratio of the probability that an event will occur vs. the probability that it will not [20]. For example, the odds ratio formula of age (≤64 years vs. ≥65 years) in logistic regression analysis of the factors related to refusing treatment was calculated as follows:

OR = [patients aged 64 years or less who did not discontinue treatment × patients aged 65 years or more who did not discontinue treatment]/[patients aged 64 years or less who did discontinue treatment × patients aged 65 years or more who did discontinue treatment].

## 3. Results

### 3.1. Patient and Clinical Characteristics

Of the 1247 BC patients studied, the average age at diagnosis was 69.15 years (SD, 12.09 years), and more than half were aged ≥65 years (64.7%). Most patients were male (72.8%), married (84.5%), and were educated at the junior or senior high school level (43.0%). Most patients lived in the north (93.3%), which reflects that the data for this study came from a medical center in Northern Taiwan; public transportation availability resulted in medical accessibility. Nearly two-thirds of patients were aged 65 years or more; as the retirement age in Taiwan is 65 years, a majority of subjects therefore reported being unemployed (66.9%). A majority of the patients had NMIBC (63.5%), had 0–I stage disease (63.5%) at diagnosis, had no disease recurrence (93.3%), and had received surgery only or surgery with immunotherapy or CT or RT or CCRT (75.9%). Of the 1247 patients, 2.1% (*n* = 26) refused treatment and 4.3% (*n* = 53) discontinued treatment (Table 1).

### 3.2. Factors Related to Refusing Treatment

A total of 26 BC patients (2.1%) reported refusing treatment. The factors associated with refusing treatment included lower level of education (odds ratio, OR, 0.855; 95% confidence interval, CI, 0.758–0.964; *p* < 0.05) and cancer stage II–IV (OR, 11.615; 95% CI 1.529–88.252; *p* < 0.05) (Table 2).

### 3.3. Reasons for Refusing Treatment

In descending order, the reasons for refusing treatment included “patient’s poor physical condition” (46.2%), “complementary and/or alternative medicine use” (38.5%), “negative treatment effects or worry about side effects” (11.5%), and “poor family support” (3.8%) (Table 3).

### 3.4. Factors Related to Discontinuing Treatment

A total of 53 BC patients (4.3%) reported discontinuing treatment. Patients were more likely to discontinue treatment if they were not living in the northern region of Taiwan (OR, 3.961; 95% CI 1.766–8.885, *p* < 0.01) or had cancer stage II–IV (OR, 3.711; 95% CI 1.401–9.831, *p* < 0.01) (Table 4).

### 3.5. Reasons for Discontinuing Treatment

Ranked in descending order, the top five reasons for discontinuing treatment included “inconvenient transportation” (49.1%), “distrust of physician ability” (17.0%), “death” (11.3%)”, “patient’s poor physical condition” (9.4%), and “negative treatment effects or worry about side effects” (7.5%) (Table 5).

## 4. Discussion

In our study, 2.1% of BC patients reported refusing treatment, a much smaller percentage than that reported in previously studies [8,9,10]. Chen et al. [8] found that a total of 3.54% (*n* = 1243) of breast cancer patients reported refusing treatment. Chiang et al. [9] surveyed 3441 colorectal cancer patients, of whom 1.98% (*n* = 68) had declined treatment (including surgery; radiotherapy, RT; CT; targeted therapy; or postoperative CT). Mehta et al. [10] found that fewer than 2% of early-stage non-small cell lung cancer patients refused recommended surgery. The difference may have been influenced by cancer stage and survival rate, as most of our subjects had 0–I cancer stage at diagnosis. Those diagnosed with 0–I cancer stage have a much better outcome than those diagnosed with II–IV cancer stage, or those with the cancers previously studied (breast, lung, or head and neck cancer). These findings suggest that a treatment plan is needed to address treatment efficacy and survival outcomes, to prevent refusal of treatment.

Results of the present study showed that BC patients with fewer years of education and those diagnosed at cancer stage II–IV were more likely to refuse treatment. These findings are similar to those of Suh et al. [7], who demonstrated that low educational status in lung cancer patients was significantly related to refusal of cancer treatment. BC patients with II–IV cancer stage are often recommended to receive radical cystectomy with ileal conduit. These therapies may lead to a permanent cystostomy following surgery, which may destroy bodily integrity and restrict social activities [21]. Gorphe et al. [22] similarly reported that advanced laryngeal cancer patients who refused a total laryngectomy did so following an insufficient response to induction CT. Chang et al. [23] also found that muscle-invasive bladder cancer patients with advanced stage cancer and those recommended to receive surgery plus RT, CT, or CCRT were more likely to refuse treatment. In the present study, more of the patients refusing treatment were aged 65 years or older and more than half were II–IV cancer stage at initial diagnosis. Understanding patient concerns and their and their families’ attitudes toward to treatment and providing relevant information can help patients understand the advantages of agreeing to life-saving treatment.

In the present study, the most common reason for refusing treatment was a concern that the treatment would worsen the patient’s condition. The most common reason for discontinuing treatment was the lack of convenient transportation. These findings agree with those of previous studies of patients with head and neck cancer [23] and those with colorectal cancer [9], which indicated that patients or their families refused treatment when they strongly agreed that the treatment-related adverse effects led to a decline in physical function and that patients’ poor physical condition might limit their ability to cope with the adverse effects of treatment. More than half of patients with BC who refused treatment were older adults, who are more likely to have comorbidities. Thus, it is important to assess each patient’s health status and potential stressors before treatment.

In the present study, 4.3% of BC patients reported discontinuing treatment, a rate lower than that reported in previous studies [12,13,15]. Dispinzieri et al. [12] revealed that a total of 9.6% of elderly breast cancer patients reported discontinuing hormone therapy. Eichler et al. [13] surveyed 1322 breast cancer patients and found that 6.3% had stopped therapy due to toxicity. Honecker et al. [15] demonstrated that a total of 57.6% of elderly men with castration-resistant prostate cancer discontinued their scheduled treatment; the main reasons were progressive disease/death in 63%, adverse events/toxicity in 22%, and withdrawal of consent in 8%. This difference in the rate of discontinuing treatment may be attributable in part to the fact that more than half of our study subjects were diagnosed with NMIBC and treated with incisionless surgery plus intravesical CT. NMIBC patients who received treatment reported fewer treatment-related adverse effects and may therefore be more compliant to treatment. Healthcare professionals should assess patients’ problems and provide care that helps to minimize the adverse effects of treatment to prevent treatment disruption.

Patients not living in the northern region of Taiwan were more likely to discontinue treatment, results consistent with those of previous research [9]. Chiang et al. [9] found that colorectal cancer patients living in a non-northern region of Taiwan were more likely to discontinue treatment. The greater likelihood of discontinuing treatment in patients living in the central, southern, or eastern regions of Taiwan may have been the result of the adverse effects of treatment, leading to a decline in physical fitness and increasing the fatigue of a long journey to the hospital for more treatment. In clinical care, healthcare providers can enhance patients’ physical activity and nutrition supplementation in order to increase their physical endurance. Access to transportation and the provision of additional travel information may help prevent such patients from terminating treatment.

No statistically significant associations were found between patients by marital status, patient age (64 years or less vs. 65 years or more), treatment refusal, or treatment discontinuation. These results did not support our research assumption [7,8,10,12,16,17]. These findings may reflect the fact that, in this population, age and spousal support do not affect whether a patient refuses or discontinues treatment. According to clinical observation, maintaining good physical functioning and a positive attitude toward to anti-cancer treatment helps patients continue to perform functional tasks, which includes activities of daily living, and cope with treatment-related adverse effects.

This study has several limitations. First, the study examined the factors associated with patients with BC refusing or discontinuing treatment using retrospective secondary database analysis, which may limit the interpretation of results. Prospective study and qualitative interviews are needed to definitively identify factors associated with refusing and discontinuing treatment. Second, we were not able to analyze patients’ initial (pre-treatment) physical functioning, which may have affected the reasons why treatment was refused or discontinued. Further studies are needed to determine the correlation between initial health status and refusing or discontinuing treatment. Finally, the data were taken from the CRD of a medical center in Northern Taiwan and may not fully account for the geographical and sociological factors at work in other countries, which may limit the generalizability and representativeness.

## 5. Conclusions

### 5.1. Conclusions

A total of 1247 patients were included in this study. We found that 2.1% of BC patients reported refusing treatment. Patients with less education and those diagnosed at cancer stage II–IV were more likely to refuse treatment. The major reasons for refusing treatment were the patient’s poor physical condition, complementary and/or alternative medicine use, negative effects of treatment or worry about side effects, and poor family support. A total of 4.3% patients reported discontinuing treatment. Patients not living in the northern region of Taiwan and those diagnosed at cancer stage II–IV were more likely to discontinue treatment. The major reasons for discontinuing treatment were lack of convenient transportation, dissatisfaction with medical care, death, the patient’s poor physical condition, and the negative effects of treatment or worry about side effects.

### 5.2. Clinical Implications

The results of this study provide a reference for clinical assessment of the reasons for refusing or discontinuing treatment in patients with BC. Healthcare providers should identify and assess cancer stage, recommend a treatment plan, and provide information to help patients make appropriate treatment decisions. Sufficient social resources and supportive care are necessary to help patients overcome the suffering encountered during the treatment period.

## Figures and Tables

**Table 1:** Demographic and clinical characteristics of patients (*n* = 1247).

Variable	Number (%)	Mean (SD)	Range
Age		69.15 (12.09)	20–104
≤64	440 (35.3)		
≥65	807 (64.7)		
Gender			
Male	908 (72.8)		
Female	339 (27.2)		
Marital status			
Unmarried	193 (15.5)		
Married	1054 (84.5)		
Education level			
None	107 (8.6)		
Elementary	398 (31.9)		
Junior and senior high	536 (43.0)		
College and above	206 (16.5)		
Occupation			
Unemployed	834 (66.9)		
Employed	413 (33.2)		
Regions of residence			
Northern Taiwan	1163 (93.3)		
Central Taiwan	56 (4.5)		
Southern Taiwan	19 (1.5)		
Eastern Taiwan	9 (0.7)		
Diagnosis			
Non-muscle invasive bladder cancer (NMIBC)	790 (63.5)		
Muscle invasive bladder cancer (MIBC)	455 (36.5)		
Cancer stage			
0–I	790 (63.5)		
II–IV	455 (36.5)		
Recurrence			
No	1163 (93.3)		
Yes	84 (6.7)		
Medical treatments			
Surgery only or surgery+ immunotherapy/CT/RT/CCRT	947 (75.9)		
CT/RT/CCRT/immunotherapy	241 (19.4)		
BST	59 (4.7)		
Refused treatment			
No	1221 (97.9)		
Yes	26(2.1)		
Discontinued treatment			
Yes	53 (4.3)		
No	1194 (95.7)		

Abbreviation: SD, standard deviation; RT, radiotherapy; CT, chemotherapy; CCRT, concurrent chemo-radiotherapy; BST, best supportive care.

**Table 2:** Logistic regression analysis of factors related to refusing treatment (*n* = 1247).

Variable	Beta	SE	Wald Test	*p*	Odds Ratio (95% CI)
Age (≤64 years vs. ≥65 years)	−0.254	0.569	0.200	0.655	0.776 (0.254–2.364)
Education level	−0.157	0.061	6.589	0.010	0.855 (0.758–0.964)
Marital status (unmarried vs. married)	0.069	0.768	0.008	0.928	1.072 (0.238–4.825)
Employment status (no vs. yes)	0.337	0.561	0.360	0.548	1.401 (0.466–4.208)
Living location (northern vs. other)	−0.028	0.777	0.001	0.971	0.973 (0.212–4.461)
Cancer stage (0–I vs. II–IV)	2.452	1.035	5.617	0.018	11.615 (1.529–88.252)
Recurrence (no vs. yes)	−0.484	1.042	0.216	0.642	0.616 (0.080–4.746)
Surgery (no vs. yes)	1.109	0.594	3.488	0.062	3.031 (0.947–9.705)
CCRT (no vs. yes)	0.279	0.790	0.124	0.724	1.321 (0.281–6.217)
Constant	−5.621	1.460	14.833	0.001	0.004 (---)

Abbreviations: SE, standard error; CI, confidence interval; RT, radiotherapy; CT, chemotherapy; CCRT, concurrent chemo-radiotherapy. 1. Chi-square = 193.534, *p* < 0.05, Nagelkerke R^2^ = 0.136. 2. Input independent variable: covariates included age (≤64 vs. ≥65), education level, marital status (unmarried vs. married), employment status (no vs. yes), living location (northern vs. other), cancer stage (0–I vs. II–IV), recurrence (no vs. yes), surgery (no vs. yes), and CCRT (no vs. yes).

**Table 3:** Reasons for refusing treatment (*n* = 26).

Rank	Reason	*n* (%)
1	Patient or the family considered the patient’s poor physical condition (chronic disease or unstable systemic disease), or difficulty in enduring any condition likely to cause physical discomfort from disease treatment (patient’s poor physical condition)	12 (46.2)
2	Selected complementary and/or alternative medicine	10 (38.5)
3	Patient or their family or friends experienced negative treatment effects and worried about the side effects of treatment	3 (11.5)
4	Poor family support	1 (3.8)

**Table 4:** Logistic regression analysis of factors related to discontinuation of treatment (*n* = 1247).

Variable	Beta	SE	Wald Test	*p*	Odds Ratio (95% CI)
Age (≤64 years vs. ≥65 years)	0.001	0.389	0.000	0.997	1.001 (0.467–2.148)
Education level	0.040	0.036	1.230	0.267	1.041 (0.970–1.117)
Marital status (unmarried vs. married)	1.364	1.023	1.779	0.182	3.913 (0.527–29.043)
Employment status (no vs. yes)	−0.201	0.419	0.231	0.631	0.818 (0.360–1.858)
Living location (northern vs. other)	1.377	0.412	11.154	0.001	3.961 (1.766–8.885)
Cancer stage (0–I vs. II–IV)	1.311	0.497	6.961	0.008	3.711 (1.401–9.831)
Recurrence (no vs. yes)	−0.708	0.743	0.908	0.341	0.493 (0.115–2.113)
Surgery (no vs. yes)	−0.076	0.352	0.046	0.830	0.927 (0.465–1.848)
CCRT (no vs. yes)	−0.921	0.763	1.459	0.227	0.398 (0.089–1.775)
Constant	−5.730	1.233	21.581	0.001	0.003 (---)

Abbreviations: SE, standard error; CI, confidence interval; CCRT, concurrent chemo-radiotherapy. 1. Chi-square = 361.964, *p* <0.05, Nagelkerke R^2^ = 0.048. 2. Input independent variables: covariates included age (≤64 vs. ≥65), education level, marital status (unmarried vs. married), employment status (no vs. yes), living location (northern vs. other), cancer stage (0–I vs. II–IV), recurrence (no vs. yes), surgery (no vs. yes), and CCRT (no vs. yes).

**Table 5:** Reasons for discontinuing treatment (*n* = 53).

Rank	Reason	*n* (%)
1	Inconvenient transportation	26 (49.1)
2	Distrust of physician ability and skills	9 (17.0)
3	Death	6 (11.3)
4	Patient or the family considered the patient’s poor physical condition (chronic disease or unstable systemic disease), or difficulty in enduring any condition likely to cause physical discomfort from disease treatment (patient’s poor physical condition)	5 (9.4)
5	Patient or their family or friends experienced negative treatment effects and worried about the side effects of treatment	4 (7.5)
6	Poor family support	1 (1.9)
7	Longer awaiting time for treatment	1 (1.9)
8	Dissatisfaction with medical care	1 (1.9)

## Data Availability

The data that support the findings of this study are available from the corresponding author. Restrictions apply to the availability of these data, which were used under license for this study. Data are available from the authors with the permission of Chang Gung Memorial Hospital Research Program in Taiwan.

## References

[1] Bray F., Ferlay J., Soerjomataram I., Siegel R.L., Torre L.A., Jemal A. (2018). Global cancer statistics 2018: GLOBOCAN estimates of incidence and mortality worldwide for 36 cancers in 185 countries. CA Cancer J. Clin..

[2] Global Cancer Observatory. https://gco.iarc.fr.

[3] Taiwan Cancer Registry 2016 Annual Report. http://crs.cph.ntu.edu.tw/.

[4] Mushtaq J., Thurairaja R., Nair R. (2019). Bladder cancer. Surgery.

[5] Danna B.J., Metcalfe M.J., Wood E.L., Shah J.B. (2016). Assessing symptom burden in bladder cancer: An overview of bladder cancer specific health-related quality of life instruments. Bladder Cancer.

[6] Van Kleffens T., Van Leeuwen E. (2005). Physicians’ evaluations of patients’ decisions to refuse oncological treatment. J. Med. Ethics.

[7] Na Suh W., Kong K.A., Han Y., Kim S.J., Lee S.H., Ryu Y.J., Lee J.H., Shim S.S., Kim Y., Chang J.H. (2017). Risk factors associated with treatment refusal in lung cancer. Thorac. Cancer.

[8] Chen S.J., Kung P.-T., Huang K.H., Wang Y.-H., Tsai W.-C. (2015). Characteristics of the delayed or refusal therapy in breast cancer patients: A longitudinal population-based study in Taiwan. PLoS ONE.

[9] Chiang T.Y., Wang C.H., Lin Y.F., You J.F., Chen J.S., Chen S.C. (2018). Colorectal cancer in Taiwan: A case-control retrospective analysis of the impact of a case management program on refusal and discontinuation of treatment. J. Adv. Nurs..

[10] Mehta R.S., Lenzner D., Argiris A. (2011). Race and health disparities in patient refusal of surgery for early-stage non-small cell lung cancer: A SEER cohort study. Ann. Surg. Oncol..

[11] Jacob L., Hadji P., Albert U.-S., Kalder M., Kostev K. (2015). Impact of disease management programs on women with breast cancer in Germany. Breast Cancer Res. Treat..

[12] Dispinzieri M., La Rocca E., Meneghini E., Fiorentino A., Lozza L., Di Cosimo S., Gennaro M., Cosentino V., Sant M., Pignoli E. (2018). Discontinuation of hormone therapy for elderly breast cancer patients after hypofractionated whole-breast radiotherapy. Med. Oncol..

[13] Eichler M., Singer S., Janni W., Harbeck N., Rack B., Augustin D., Wischnik A., Kiechle M., Ettl M.J., Scholz C. (2016). Pretreatment quality of life, performance status and their relation to treatment discontinuation and treatment changes in high-risk breast cancer patients receiving chemotherapy: Results from the prospective randomized ADEBAR trial. Breast Cancer.

[14] Freedman R.A., Fedewa S.A., Punglia R.S., Lin C.C., Ward E.M., Jemal A., Sineshaw H.M. (2015). Factors associated with radiation therapy incompletion for patients with early-stage breast cancer. Breast Cancer Res. Treat..

[15] Honecker F., Wedding U., Kallischnigg G., Schroeder A., Klier J., Frangenheim T., Weißbach L. (2018). Risk factors for unplanned discontinuation of scheduled treatment in elderly patients with castration-resistant prostate cancer: Results of the IBuTu study. J. Cancer Res. Clin. Oncol..

[16] Lee J.A., Lee N.K., Yoon W.S., Yang D.S., Kim C.Y., Lee S.R., Seong H.J. (2016). Treatment interruption during radiation therapy: Experience at a single institution in the Republic of Korea. Asia Pac. J. Clin. Oncol..

[17] Puckett L.L., Luitweiler E., Potters L., Teckie S. (2017). Preventing discontinuation of radiation therapy: Predictive factors to improve patient selection for palliative treatment. J. Oncol. Pr..

[18] Sethi R.A., Stamell E.F., Price L., DeLacure M., Sanfilippo N.J. (2010). Head and neck radiotherapy compliance in an underserved patient population. Laryngoscope.

[19] World Health Organization (2010). International Statistical Classification of Diseases and Related Health Problems.

[20] Altman D.G. (1991). Practical Statistics for Medical Research.

[21] Arcangeli G., Strigari L. (2015). Radical cystectomy versus organ-sparing trimodality treatment in muscle-invasive bladder cancer: A systematic review of clinical trials. Crit. Rev. Oncol..

[22] Gorphe P., Matias M., Blanchard P., Even C., Ferte C., Tao Y., Temam S., Bidault F., Janot F. (2017). Outcomes following laryngectomy refusal after insufficient response to induction chemotherapy. Laryngoscope.

[23] Chang Y.L., Lee S.C., Liao C.T., Wang C.H., Lin Y.F., Chen S.C. (2019). Factors impacting on discordance with treatment plan in head and neck cancer patients: A retrospective, population-based cohort study. Support. Care Cancer.

